# Learning Stress, Involvement, Academic Concerns, and Mental Health among University Students during a Pandemic: Influence of Fear and Moderation of Self-Efficacy

**DOI:** 10.3390/ijerph191610151

**Published:** 2022-08-16

**Authors:** Jian Yang, Ling Xiang, Shaobang Zheng, Huijing Liang

**Affiliations:** 1 School of Journalism and Communication, Guangzhou University, Guangzhou 510006, China; 2 School of Shipping Economics and Trade, Guangzhou Maritime University, Guangzhou 510725, China

**Keywords:** COVID-19, learning stress, involvement, academic concerns, psychological well-being, self-efficacy

## Abstract

COVID-19 has had a profound impact on the mental health and well-being of students. An effective method that can enable students to cope with difficult times is to help them realize their inner potential. Following the stimulus-organism-response model, this study developed a theoretical framework that deepens our understanding of an environmental stimulus (fear of COVID-19) that is experienced by students; struggle within the organism (learning stress, learning involvement, and academic concerns); and the psychological response (psychological well-being). The findings clarified how the fear of COVID-19 affects the psychological well-being of university students and revealed the moderate role of academic self-efficacy in this process. Some systematic practical advice was provided to higher education institutions to develop effective interventions to protect the mental health of college students and establish strategies to promote their inner potential.

## 1. Introduction

COVID-19 is a highly contagious, unprecedented pandemic that affects a broad area of the world. There is no doubt that the pandemic has affected people of all ages, but college students are particularly vulnerable. According to a report that was prepared by UNESCO, approximately 75% of students in higher education institutions felt anxious and uneasy as a result of the suspension of their studies [1]. Owing to social distancing and the trend towards online learning, university students are obtaining less support from friends, classmates, professors, and family, which can contribute to their mental health problems [2,3]. Students’ loss of jobs and concerns for their financial situation, future education, and careers led to many students experiencing personal financial difficulties. These factors have been interpreted as the cause of many emotional problems [4,5]. Research has shown that psychological stress that is caused by the COVID-19 pandemic and social distancing measures that lead to mental health issues such as mood disturbances, irritability, and insomnia can further lead to other health problems [6,7]. As a result of the COVID-19 pandemic, the academic landscape has been transformed and students have faced daunting challenges when taking courses [8]. Therefore, the mental health of university students has become an important public health problem [9], and there is an emerging need to investigate the factors that are responsible for mental health problems in the student population [10].

Psychological well-being is an important indicator of mental health [11], and it has been a frequently raised issue in this pandemic. Scholars believe that the ability to experience high levels of well-being is possible despite excessive mental health problems [12]. Current research on education during the pandemic mainly focuses on how to solve external issues to improve the mental health of students. For instance, Islam, et al. [13] indicated that poor psychological well-being is associated with problematic smartphone use and social media use. In this situation, a significant impact is made on the academic concerns of university students by the support that is provided by higher education institutions and faculty members [14]. Furthermore, there is a correlation between low psychological well-being and environmental stimuli, such as economic downturns, transportation disruptions, and lockdown regulations, all of which lead to low psychological well-being [15]. However, few studies have explored ways to realize the internal potential of students to improve psychological well-being. Many scholars noted that rather than providing resources to students in the event of a negative mental health situation, it is more effective for educational institutions to help students realize their inner potential so that they can adapt well to challenging times [16,17]. 

On the other hand, the stimulus-organism-response (SOR) model is often used to investigate the learning problems of students facing a special environment. Such studies often use behavioral outcomes as response variables [15,18]. However, with further research, the understanding of response variables should be on two levels: the response at the behavioral level and the response at the psychological level [19,20]. Few studies on education have explored responses at the psychological level. To fill the above two research gaps, this research uses fear of COVID-19 as the stimulus variable; learning stress, learning involvement, and academic concerns as the organism variable; and psychological well-being as the psychological level response variable to construct a research model. The objective of this study is to examine the association between environmental stimuli (fear of COVID-19); struggle within the organism (learning stress, learning involvement and academic concerns); and psychological response (psychological well-being) in the university student population and verify the moderating role of academic self-efficacy in the relationship.

## 2. Literature Review and Hypothesis Development

### 2.1. Fear of COVID-19

To prevent the spread of the viral disease, the imposition of lockdown measures became inevitable. Schools and universities were the first to opt for complete closure in many countries. During this period, the behavior and psychology of university students were greatly affected, and they suffered from academic anxiety and fear [15]. A recent study found that university students reported experiencing a variety of psychosomatic symptoms that were related to an infection with coronavirus, such as increased pulse rate, heart palpitations, and insomnia [21]. In most cases, university students’ fear of the virus is not based on the physical danger the virus poses but instead stems from anxiety about their future careers and education as well as academic problems that are associated with personal illness [22,23]. 

The SOR model that was proposed by Woodworth and Marquis [24] defines a stimulus as the external force that influences the psychological state of a person. Ypsilanti, et al. [25] found that affective responses are an effective way to assess the stimulus that is associated with COVID-19, such as feelings of fear, sadness, and loneliness. Evidence of these fears, caused by a perception of threatening stimuli, has already been found in previous pandemic research [26,27]. Therefore, the present study used fear of COVID-19 as the stimulus construct in the SOR model to measure the stimulus to university students.

Recent studies have found a significant association between fear and stress [28,29]. Based on research that was conducted on student groups, these stress problems are especially acute during the online learning period, when students are concerned about the failure and academic year loss of e-learning [30]. Most likely, the reason for this is that the shift to online learning affected the academic performance of students and the increased workload caused plenty of intellectual fatigue [31]. Owing to a lack of guidance from their teachers, students lost interest in attending online classes because of the lack of benefits from online learning [32]. As a means to improve the quality of life for students, previous studies suggested that universities should encourage more co-curricular and extracurricular activities among students [33], because students who display negative emotions such as distress and depression often face learning difficulties and have low involvement in class [34]. 

Taking into account the discussion above, we suggest the following hypotheses:

**Hypothesis** **1** **(H1).** *Learning stress is positively impacted by the fear of COVID-19.*

**Hypothesis** **2** **(H2).** *Learning involvement is negatively impacted by the fear of COVID-19.*

**Hypothesis** **3** **(H3).** *Academic concerns are positively impacted by the fear of COVID-19.*

### 2.2. Learning Stress, Learning Involvement, and Academic Concerns 

In the context of education, stress is a subjective assessment of the level of mental tension that is experienced by students while participating in educational activities [35]. The history of stress research that is related to learning suggests that stress is a double-edged sword for students. Stress has a positive effect on memory only at the moderate level [36,37]. Suppose a student establishes aversive interactions with class participation or the given course assignments. In that case, the student will have strong mental tension and worry about not being able to meet the required study requirements [38]. Studies that are related to learning stress during pandemics are often consistent with the negative effects of stress. The sudden emergence of the pandemic and the forced e-learning that has left students panicking and ill-prepared may exacerbate their perceived stress levels in all aspects of learning [39].

Although e-learning offers students the opportunity to acquire valuable information and knowledge, there is doubt about whether this knowledge and information can be transformed into academic ability. It is critical to create conditions that motivate and inspire students to engage in educationally relevant activities when improving student learning and development [40]. It is widely accepted that involvement contributes to improved student effort and better learning outcomes [40,41]. The concept of involvement refers to a person’s perception that they are relevant to a certain object based on their inherent values, needs, and interests [42]. In the education context, involvement in learning is a fundamental component of the cognitive and emotional development of students [43]. The learning involvement of students is determined by how much physical and psychological effort they devote to the academic process [44]. It is more predictive of personal development and learning outcomes when students devote time and effort to educational activities [45]. As students devote more time and energy to their learning processes, their self-achievement and satisfaction with their learning experiences are enhanced [40].

The sense of academic loss and dissatisfaction that university students developed due to the pandemic has led to concerns about their academic performance [46]. There are various academic concerns that restrict students’ capacity to maintain concentration during e-learning [14]. Previous studies repeatedly indicated that student involvement is critical to their academic success [47,48]. In other words, students who have more learning involvement are less likely to perceive learning difficulties and be concerned about their academics. Identifying stressors is also another way to reduce academic concerns among university students [49].

From the perspective of the SOR model, the perceptions, feelings, and thoughts of an individual can be described as an ‘organism’ [50]. In the study of education, organism refers to the emotional response of the student to the COVID-19 crisis and represents the internal emotion and psychological processes that are triggered by the stimulus [15]. Based on the concept of learning stress, involvement, and academic concerns, it is reasonable for these three theoretical constructs to be regarded as organisms. Taking into account the discussion about the relationship between learning stress, involvement, and academic concerns, we suggest the following hypotheses:

**Hypothesis** **4** **(H4).** *Learning stress has a positive impact on academic concerns.*

**Hypothesis** **5** **(H5).** *Learning involvement has a negative impact on academic concerns.*

### 2.3. Psychological Well-Being

Psychological well-being is defined as the ability of a person to function psychologically, develop and maintain positive relationships, feel autonomous, accept themselves, grow personally, find meaning in life, and feel empathy towards others [51]. To realize one’s full potential, one must be in good psychological well-being [52]. This is also true for students with regard to academic achievement [53]. The World Health Organization (WHO) Well-being Index is often used in education studies to measure psychological well-being based on the WHO definition that psychological well-being is a state of inner fulfilment that allows individuals to realize their potential [16,54,55]. In terms of the impact on educational outcomes, psychological well-being refers to the development of one’s true potential and is viewed as the consequence of a life well lived as well as an important component of students’ success in college or university [56].

Owing to lockdowns during the COVID-19 pandemic, lack of certainty [57], perceived stress [17], and concern for the future [58] continue to affect the psychological well-being of students. The shift to online learning as a result of the suspension of face-to-face classes has negatively affected the psychological well-being of students because of the decline in student involvement [59]. Involvement in campus experiences has been demonstrated to be positively correlated with psychological well-being [60]. Particularly, co-curricular involvement is associated with psychological well-being on several levels, such as personal growth, positive relationships with others, and purpose in life [61]. 

In the SOR framework, response is defined as individuals’ reactions that are affected by environmental factors [20], including behavioral [15] and psychological reactions [19]. In this study, psychological well-being is viewed as a construct of psychological reactions. Taking into account the discussion above, we suggest the following hypotheses:

**Hypothesis** **6** **(H6).** *Psychological well-being is negatively impacted by learning stress.*

**Hypothesis** **7** **(H7).** *Psychological well-being is positively impacted by learning involvement.*

**Hypothesis** **8** **(H8).** *Psychological well-being is negatively impacted by academic concerns.*

### 2.4. Academic Self-Efficacy

A person’s ability to adapt and function under adverse circumstances can be characterized as their resilience [62,63]. Resilience also helps students cope with adversity as they face the impact of the pandemic. According to Morales-Rodríguez [28], students’ fear of COVID-19 has an inverse relationship with their use of cognitive restructuring coping strategies, while variable resilience has a direct positive relationship with problem-solving coping strategies. Psychological resilience also plays a significant role in predicting their fear of COVID-19 [64]. However, self-efficacy as an effective path to achieving resilience [65] has rarely been used to explore the impact on the academic performance of students during the pandemic. According to Bender and Ingram [65], resilience depends on some belief that one can exert control over the environment. Consequently, self-efficacy is likely to encourage resilient behaviors and attitudes.

As defined by social cognitive theory, self-efficacy refers to the belief in the ability that an individual will be able to achieve a desired outcome in the future [66]. It is widely believed that self-efficacy is one of the most important non-intelligent factors that will encourage academic motivation, processes, and outcomes [67], and the relationship between self-efficacy and academic performance has been consistently demonstrated to be positive in many studies [68,69]. The possible reason for this finding is that self-efficacious students are able to regulate and self-monitor their impulses and persevere even in difficult situations [70]. Given the uncertainty that was associated with COVID-19, self-efficacy became significantly more important in mobilizing the resources that were required for success [71]. As a result, schools should implement policies that will enhance the self-confidence and self-efficacy levels of students to ensure optimal learning outcomes [72].

Taking into account the discussion above, we suggest the following hypotheses:

**Hypothesis** **9** **(H9).** *Academic self-efficacy has a negatively moderating impact on the correlation between fear of COVID-19 and learning stress.*

**Hypothesis** **10** **(H10).** *Academic self-efficacy has a negatively moderating impact on the correlation between fear of COVID-19 and learning involvement.*

**Hypothesis** **11** **(H11).** *Academic self-efficacy has a negatively moderating impact on the correlation between fear of COVID-19 and academic concerns.*

**Figure 1:**
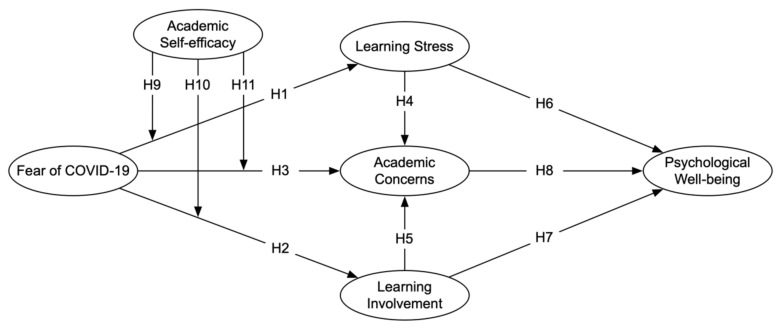
Conceptual framework.

Based on Hypotheses 1 to 11 that are established above, Figure 1 presents the theoretical framework of this study. From the perspective of the SOR model, fear of COVID-19 is considered as the stimulus; learning stress, learning involvement, and academic concerns are considered as the organism; and psychological well-being is considered as the response.

## 3. Methodology

In order to understand the relationship between fear of COVID-19 and psychological well-being among university students and verify every hypothesis based on the SOR theory, a quantitative research methodology with partial least squares structural equation modelling (PLS-SEM) was employed. As a statistical technique, PLS-SEM has been demonstrated to be effective in the analysis of complex or exploratory research models and is particularly suitable for analyzing the effects of moderating variables on the relationship between two constructs [73,74]. Considering the complex structure of our research model, which includes eight direct effect hypotheses and three moderating effect hypotheses, PLS-SEM offers the best fit for this study. 

According to the suggestion that Hair Jr, et al. [75] offered, two parts of the data analysis process were carried out, including the evaluation of the measurement model and the structure model.

### 3.1. Survey Instrument and Measurement Items

There were two sections that were included in the survey instrument. 

In Section 1, 35 items that were adapted from existing studies represented the six constructs in the research model. Each item was modified to be suitable for evaluating the learning status of the student. As the measurement items were written in English, the back-translation method was required [76]. Compared with the original scale, some items were deleted due to low factor loading. Fear of COVID-19 (7 items) was adapted from Ahorsu, et al. [77]. Learning stress (6 items) was adapted from Yang and Chen [78], Rabaglietti, et al. [79], and Cohen, et al. [80]. The original statement ‘during the last month’ was changed to ‘Since the period of the COVID-19 pandemic’. A sample item would be as follows: ‘Since the period of the COVID-19 pandemic, how often have you felt nervous and stressed in learning?’ Learning involvement (6 items) was adapted from Yang, Zhou, and Cheng [44] and Jiang, et al. [81]. A sample item would be as follows: ‘I think learning is unimportant/important’. Academic concerns (3 items) were adapted from Al-Maskari, Al-Riyami, and Kunjumuhammed [14]. Psychological well-being (4 items) was adapted from the World Health Organization [82]. Academic self-efficacy (9 items) was adapted from Roche et al. [83]. The following is a sample item: ‘I can learn what is being taught in class this semester’. To assess fear of COVID-19, academic concerns, psychological well-being, and academic self-efficacy, we used a seven-point Likert scale from ‘strongly disagree’ to ‘strongly agree’. To assess learning stress, we used a seven-point Likert scale that was rated from ‘never’ to ‘always’. To assess learning involvement, we used a seven-point Likert scale that was rated from ‘negative attitude’ to ‘positive attitude’.

In Section 2, the four demographic questions included gender, age, grade, and major.

All the measurement items are described in detail in Appendix A.

### 3.2. Data Collection

The survey was aimed at undergraduate students who were studying online as a result of the quarantine measures in mainland China. Questionnaire screening questions were used to verify respondents’ identities. As the data were collected during a time when social distancing was appropriate, an online survey was the most appropriate means to collect data; it has been proven reliable to collect data using this method in COVID-19 studies [84]. Since March 2022, the number of infections in mainland Chinese cities such as Shanghai and Shenzhen has soared because of the COVID-19 variant virus. University students who had resumed their normal studies were once again hit by the pandemic. Therefore, the questionnaire data of this study were collected from March 15 to April 30, 2022. A link to an online questionnaire was sent via social media. Initially, the questionnaire was distributed through WeChat to the personal networks of the researchers in mainland China. Subsequently, survey respondents were encouraged to share the survey link with their friends and classmates. There was no incentive scheme in place. In total, we were able to use 245 questionnaires. According to the suggestion that the sample-to-item ratio should not be less than 5-to-1 [85], the sample size that was obtained in this study meets the minimum sample requirements.

## 4. Results

### 4.1. Sampling Profile

Information on the respondents’ demographics that were derived from the formal investigation stage are presented in Table 1. Male respondents accounted for 48.2% and female respondents for 51.8%. Respondents that were aged 20 and 21 made up the majority. The survey participants were mainly freshmen, sophomores, and juniors. A total of 41.6% of respondents majored in Social Science and 42.9% majored in Science & Engineering. 

### 4.2. Evaluation of the Measurement Model

This section contains the evaluation results that were used primarily to demonstrate the reliability, convergent validity, and discriminant validity of the measurement model. 

According to Hair, et al. [86], there are two indicators for judging whether the measurement model has acceptable reliability, namely Cronbach’s α values and CR values that are both greater than 0.7. The results of reliability and convergent validity are presented in Table 2. Cronbach’s α values ranging from 0.891 to 0.949 were obtained. The CR values were within the range of 0.917 to 0.959. The result indicates that the reliability of the measurement model is satisfactory.

For the acceptable convergent validity criterion, there are also two indicators [86], namely factor loadings (from 0.703 to 0.930) and AVE values (from 0.592 to 0.827) both above the threshold value. As a result, the measurement model has adequate convergent validity.

Table 3 shows the computational results of the two techniques for verifying discriminant validity. They are Fornell–Larcker criterion and HTMT analysis. The Fornell–Larcker criterion requires that the correlation between constructs must be less than the square root of the AVE on each construct [87]. A satisfying result of the Fornell–Larcker criterion can be seen in Table 3. HTMT analysis suggested that all HTMT ratios of constructs were below 0.85 [88]. In Table 3, the values above the bold fonts are the HTMT ratios. All HTMT values ranged from 0.125 to 0.498, again with satisfactory results.

### 4.3. Structural Model Evaluation

Table 4 shows the R^2^ values and Q^2^ values that were used to verify whether the data can be accurately predicted by the structural model. The minimum acceptable value for R^2^ is 0.1 [89] and the minimum acceptable value for Q^2^ is 0 [86]. According to R^2^ values ranging from 0.119 to 0.292 and Q^2^ values ranging from 0.071 to 0.231, it can be proved that the structural model can accurately predict the data.

Bootstrapping resampling (5000 resamples) was used to assess the statistical significance of the variables. The results are summarized in Table 5. Fear of COVID-19 is significantly associated with learning stress, learning involvement, and academic concerns (β = 0.241, *p* < 0.001; β = −0.176, *p* < 0.01; β = 0.229, *p* < 0.001), thus supporting H1, H2, and H3. Learning stress and learning involvement have significant relationships with academic concerns (β = 0.187, *p* < 0.01; β = −0.154, *p* < 0.05), thus supporting H4 and H5. Learning stress, learning involvement, and academic concerns have significant relationships with psychological well-being (β = −0.184, *p* < 0.05; β = 0.181, *p* < 0.05; β = −0.192, *p* < 0.01), thus supporting H6, H7, and H8.

The ƒ^2^ values are also used as part of the hypothesis testing to assess the quality of the hypothesis. Accordingly, all the supported hypotheses in Table 5 have an ƒ^2^ value greater than 0.02, indicating that various exogenous factors influence their corresponding endogenous factors in a significant way [75]. Furthermore, all VIFs in Table 5 are below 5, ranging from 1.011 to 1.23, indicating that the multicollinearity issue was not present [75].

### 4.4. Moderating Effect

**Figure 2:**
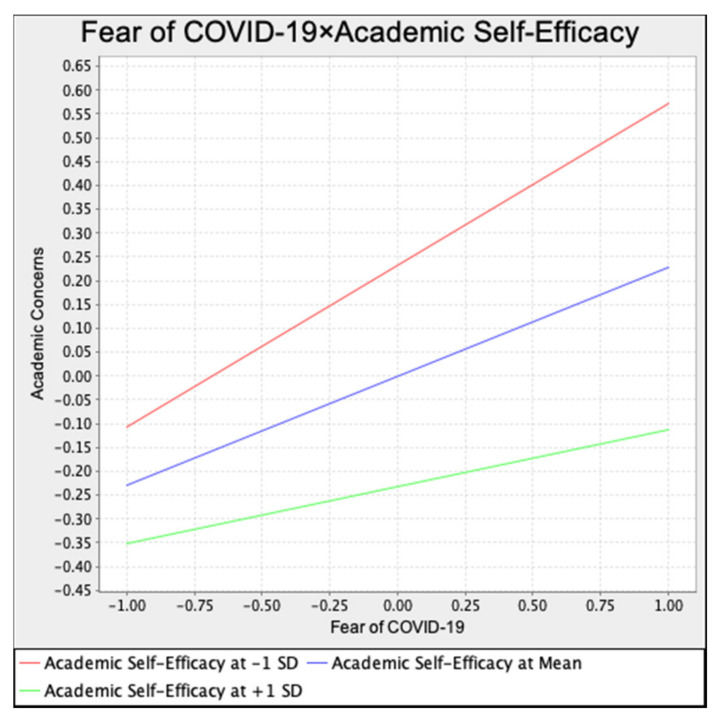
Simple slope test.

The interactive effect of academic self-efficacy and fear of COVID-19 is significantly associated with academic concerns (β = −0.11, *p* < 0.01), supporting the moderating effect of academic self-efficacy. Consequently, H11 is confirmed. According to the simple slope analysis that is shown in Figure 2, among students with the same level of fear, students who have a higher level of academic self-efficacy have lower academic concerns while the COVID-19 pandemic was underway. Given the smoother slope of the high academic self-efficacy line (the green one), academic self-efficacy has a negative moderating effect.

## 5. Discussion

The high infectivity of the COVID-19 variant virus has made the academic life of students full of uncertainty, and the original return to normal study life may be transformed to online learning in a short period. As students are afraid that the virus will affect their learning progress and thus affect their academic performance, they have a lot of fears about the virus [22,23]. The current study confirms the existence of this fear in the student group, along with its negative impact on the psychological state of students during learning. It can be predicted that the mutual influence of fear and psychological state of students can easily cause students to have psychological difficulties.

This study demonstrates how the stimuli that are brought about by environmental changes during the pandemic affect the psychological state of college students during their learning process. The study results confirm H1 to H3, indicating that university students with higher levels of fear of COVID-19 have greater learning stress (H1), lower levels of learning involvement (H2), and greater academic concerns (H3). These results are consistent with previous research that environmental changes during the pandemic are indeed detrimental to the learning status of college students [29,32].

This study also shows the psychological struggle of students in their academic life during the pandemic. Learning stress as a negative psychological activity and learning involvement as a positive psychological activity can both have effects on academic concerns. Students who were more stressed about their studies had higher levels of academic concerns (H4). Conversely, students with higher learning involvement had fewer academic concerns (H5). The results of this study support and integrate the findings of previous studies about learning stress and involvement [47,48,49]. It can be expected that the proportion of learning stress and involvement in the psychological activities of students affects the level of their academic concerns.

With regard to the mental health problems of students, their learning stress, learning involvement, and academic concerns are significantly related to their psychological well-being. Students with high levels of psychological well-being showed low levels of learning stress (H6), high levels of learning involvement (H7), and low levels of academic concerns (H8). The results of this study support previous findings [17,58,60] and integrate relevant conclusions into the psychological research of students during the pandemic period.

On the whole, based on the SOR model, the eight research hypotheses that were formed by fear of COVID-19, learning stress, learning involvement, academic concerns, and psychological well-being have been verified.

As an independent variable, the moderating effect of self-efficacy shows whether the internal potential of students can resist the negative effects that are brought by environmental stimuli. Students with high self-efficacy can exert their self-potential and psychological adjustment ability as well as reduce the influence of their fear of COVID-19 on academic concerns. In other words, under the same fear level, students with high self-efficacy have fewer academic concerns (H11). It is not difficult to understand that because students with high self-efficacy are able to proactively adjust resources and persevere through difficulties, their self-confidence reduces the increasing trend of academic concerns. This result is in line with previous studies [70,71]. However, at the same level of fear, self-efficacy cannot reduce the negative effects of environmental stimulus on learning stress and learning involvement; H9 and H10 are not supported. The most likely explanation is that the high stress and low involvement in learning that were caused by the external environmental factors during the pandemic have far exceeded the internal adjustment ability of the students.

## 6. Conclusions and Implications

In this study, a theoretical framework was developed based on the SOR model to improve our understanding of an environmental stimulus (fear of COVID-19); the struggle within the organism (learning stress, learning involvement, and academic concerns); psychological response (psychological well-being); and internal potential (self-efficacy) in an era of COVID-19. There were 11 hypothesized relationships, 9 of which were supported. The findings may contribute to the literature on the mental health of students during the pandemic and provide education practitioners with valuable insights into the phenomenon.

### 6.1. Theoretical Implications

Firstly, this study established a research framework that was based on the SOR model and focused on students’ mental health problems that were caused by environmental changes and stimuli. The results of this study extended the application of the SOR model in the psychological response and supported the previous research that the concept of response should include both behavioral and psychological responses [19].

This study likewise took both positive and negative influencing factors into account when examining academic concerns, namely learning involvement and learning stress, which compensated for the inadequacy of the existing predictors. The results show that both learning engagement and learning stress have significant effects on academic worry, which indicates that the formation of academic concerns is the result of the students’ internal regulation mechanisms. From the perspective of the formation of students’ psychological well-being during the pandemic period, academic concern is a crucial influencing factor (β = −0.192, *p* < 0.01), which enhances the value of academic concerns theory in the study of students’ mental health.

Finally, this study established self-efficacy as a moderating variable in the research model, thereby expanding the role of self-efficacy in the formation of mental health. In the context of mental health, self-efficacy not only plays a direct or indirect role in influencing [64] but also in regulating. The results show that self-efficacy alleviates the rising trend of academic concerns, which deepens the understanding of self-efficacy in student mental health research during the pandemic period.

### 6.2. Practical Implications

In this study, learning involvement and self-efficacy were identified as two critical factors that positively affect the mental health of students. It is from this point that educators can define strategies for providing support to students. Educators should consider comprehensive, accurate feedback, and assessment of students’ learning performance, as this teaching method helps students develop a greater sense of self-efficacy and a better sense of success [90]. The criteria for assessment must be well thought out, as this is crucial not only for students’ achievement but also for the development of their mental health. Poor assessment measures may impair the self-efficacy of students and affect their subsequent performance [91]. Increased self-efficacy will help students reduce academic concerns while enhancing their belief in their ability to resist distractions and focus on learning, thereby improving their learning involvement. In the face of the pandemic, it seems that online learning is inevitable, and the establishment of online discussion boards may be beneficial for timely teaching feedback and interaction between teachers and students. In addition, educators can develop students’ ability to control the use of e-learning technologies. These strategies can be used to improve the self-efficacy and self-regulation of students in an online learning environment [92].

## 7. Limitations and Future Studies

As a result of social distancing restrictions, face-to-face surveys were not possible and all surveys were conducted online, thus affecting the sample size and representativeness. Future studies may consider increasing the sample size or adopting probability sampling methods [93,94]. During a pandemic, countries adopt very different strategies for pandemic prevention; Western countries may adopt a more relaxed pandemic prevention strategy. It is important to conduct comparative studies on the same subject across different geographical areas, which will reduce the bias that can result from differences in culture, ethnicity, and immunization strategies. This study only introduces variables of self-efficacy when studying personal resilience, and more related variables, such as psychological flexibility [95] and self-compassion [96], can be explored in future studies.

## Figures and Tables

**Table 1:** Demographic information of the participants.

Demographic	Categories	Frequency	Percentage
Gender	Male	118	48.2%
	Female	127	51.8%
Age	18 or below	18	7.3%
	19	41	16.7%
	20	67	27.4%
	21	71	29.0%
	22	37	15.1%
	23 or above	11	4.5%
Grade	Freshman	63	25.8%
	Sophomore	77	31.4%
	Junior	77	31.4%
	Senior	28	11.4%
Major	Social Science	102	41.6%
	Science & Engineering	105	42.9%
	Art	38	15.5%

**Table 2:** Results of reliability and convergent validity.

Construct	Items	Loading	Standard Deviation	Cronbach’s α	CR	AVE
Fear of COVID-19 (FCV)				0.909	0.928	0.647
	FCV_1	0.859	1.479			
	FCV_2	0.857	1.533			
	FCV_3	0.710	1.403			
	FCV_4	0.783	1.741			
	FCV_5	0.805	1.552			
	FCV_6	0.810	1.389			
	FCV_7	0.800	1.589			
Learning Stress (LS)				0.891	0.917	0.650
	LS_1	0.776	1.292			
	LS_2	0.886	1.169			
	LS_3	0.872	1.156			
	LS_4	0.713	1.132			
	LS_5	0.735	1.111			
	LS_6	0.838	1.274			
Learning Involvement (LIN)				0.949	0.959	0.797
	LIN_1	0.879	1.448			
	LIN_2	0.874	1.461			
	LIN_3	0.876	1.477			
	LIN_4	0.908	1.435			
	LIN_5	0.903	1.336			
	LIN_6	0.917	1.386			
Academic Concerns (AC)				0.895	0.935	0.827
	AC_1	0.874	1.643			
	AC_2	0.950	1.841			
	AC_3	0.903	1.694			
Psychological Well-Being (PWB)				0.907	0.935	0.781
	PWB_1	0.893	1.461			
	PWB_2	0.840	1.432			
	PWB_3	0.930	1.461			
	PWB_4	0.871	1.573			
Academic Self-Efficacy (ASE)				0.914	0.929	0.592
	ASE_1	0.708	1.413			
	ASE_2	0.794	1.457			
	ASE_3	0.825	1.425			
	ASE_4	0.824	1.436			
	ASE_5	0.718	1.364			
	ASE_6	0.733	1.439			
	ASE_7	0.797	1.343			
	ASE_8	0.809	1.403			
	ASE_9	0.703	1.422			

**Table 3:** Discriminant Validity.

Construct	FCV	LS	LIN	AC	PWB	ASE
Fear of COVID-19 (FCV)	**0.805**	0.277	0.211	0.362	0.141	0.125
Learning Stress (LS)	0.263	**0.806**	0.333	0.387	0.327	0.250
Learning Involvement (LIN)	−0.209	−0.316	**0.893**	0.341	0.315	0.319
Academic Concerns (AC)	0.331	0.357	−0.320	**0.909**	0.343	0.384
Psychological Well-Being (PWB)	−0.126	−0.310	0.300	−0.315	**0.884**	0.498
Academic Self-Efficacy (ASE)	−0.099	−0.248	0.308	−0.362	0.453	**0.769**

Note: The square root of the AVE is shown by bold fonts, HTMT ratios are the values above the bold fonts and correlations are the values below the bold fonts.

**Table 4:** Determination coefficient and predictive correlation.

Latent Variable	R-Square	Q-Square
Learning Stress	0.119	0.071
Learning Involvement	0.143	0.107
Academic Concerns	0.292	0.231
Psychological Well-Being	0.172	0.118

**Table 5:** Analysis of hypotheses.

Hypothesis	β	T	*p*	ƒ^2^	VIF	Result
H1: Fear of COVID-19 → Learning Stress						
	0.241	3.718	0.000 ***	0.065	1.011	Accept
H2: Fear of COVID-19 → Learning Involvement						
	−0.176	2.628	0.009 **	0.036	1.011	Accept
H3: Fear of COVID-19 → Academic Concerns						
	0.229	3.798	0.000 ***	0.068	1.095	Accept
H4: Learning Stress → Academic Concerns						
	0.187	2.791	0.005 **	0.041	1.197	Accept
H5: Learning Involvement → Academic Concerns						
	−0.154	2.192	0.028 *	0.027	1.230	Accept
H6: Learning Stress → Psychological Well-Being						
	−0.184	2.346	0.019 *	0.034	1.209	Accept
H7: Learning Involvement → Psychological Well-Being						
	0.181	2.547	0.011 *	0.034	1.175	Accept
H8: Academic Concerns → Psychological Well-Being						
	−0.192	2.736	0.006 **	0.037	1.213	Accept
H9: Fear of COVID-19 × Academic Self-Efficacy → Learning Stress						
	−0.008	0.124	0.902	0.000	1.012	Reject
H10: Fear of COVID-19 × Academic Self-Efficacy → Learning Involvement						
	−0.114	1.765	0.078	0.019	1.012	Reject
H11: Fear of COVID-19 × Academic Self-Efficacy → Academic Concerns						
	−0.110	2.360	0.018 **	0.020	1.032	Accept

Note: *** *p* < 0.001; ** *p* < 0.01; * *p* < 0.05.

## Data Availability

The data that are presented in this study are available on request from the corresponding author. The data are not publicly available due to privacy issues.

## Appendix A

**Table A1:** Measurement Items.

**Fear of COVID-19 (FCV) Adapted from Ahorsu, Lin, Imani, Saffari, Griffiths, and Pakpour [77]**	
FCV_1	I am most afraid of the novel coronavirus.
FCV_2	It makes me uncomfortable to think about the novel coronavirus.
FCV_3	My hands become sweaty when I think about COVID-19.
FCV_4	I am afraid of losing my life because of COVID-19.
FCV_5	When watching news and stories about the novel coronavirus on social media or any other media (i.e., TV, radio), I become nervous or anxious.
FCV_6	I cannot sleep because I am worried about getting the novel coronavirus.
FCV_7	My heart races or palpitates when I think about getting COVID-19.
**Learning Stress (LS) adapted from Cohen, Kamarck and Mermelstein [80]; Yang and Chen [78]; and Rabaglietti, Lattke, Tesauri, Settanni, and De Lorenzo [79]**	
LS_1	Since the period of the COVID-19 pandemic, how often have you felt nervous and stressed in learning?
LS_2	Since the period of the COVID-19 pandemic, how often have you dealt successfully with irritating study hassles?
LS_3	Since the period of the COVID-19 pandemic, how often have you felt that you were effectively coping with important changes that were occurring in learning?
LS_4	Since the period of the COVID-19 pandemic, how often have you felt confident about your ability to handle your learning problems?
LS_5	Since the period of the COVID-19 pandemic, how often have you been able to control irritations in your study?
LS_6	Since the period of the COVID-19 pandemic, how often have you felt that you were on top of things in learning?
**Learning Involvement (LIN) adapted from Yang, Zhou, and Cheng [44] and Jiang, Chan, Tan, and Chua [81]**	
LIN_1	I think learning is unimportant/important.
LIN_2	I think learning is means nothing/means a lot.
LIN_3	I think learning is worthless/valuable.
LIN_4	I think learning is boring/interesting.
LIN_5	I think learning is unexciting/exciting.
LIN_6	I think learning is unappealing/appealing.
**Academic Concerns (AC) adapted from Al-Maskari, Al-Riyami, and Kunjumuhammed [14]**	
AC_1	You were concerned that you would not be able to complete the academic semester.
AC_2	My university/college workload has significantly increased during the pandemic.
AC_3	You were concerned that you would not be able to graduate on time.
**Psychological Well-Being (PWB) adapted from the World Health Organization [82]**	
PWB_1	I have felt cheerful and in good spirits.
PWB_2	I have felt calm and relaxed.
PWB_3	I have felt active and vigorous.
PWB_4	I woke up feeling fresh and rested.
**Academic Self-Efficacy (ASE) adapted from Roche, Manzi, Ndubuizu, and Baker [83]**	
ASE_1	I can learn what is being taught in class this semester.
ASE_2	Once I have decided to accomplish something that is important to me, I keep trying to accomplish it, even if it is harder than I thought.
ASE_3	I am confident that I will achieve the goals that I set for myself.
ASE_4	When I am struggling to accomplish something difficult, I focus on my progress instead of feeling discouraged.
ASE_5	I believe hard work pays off.
ASE_6	My ability grows with effort.
ASE_7	I believe that the brain can be developed like a muscle.
ASE_8	I think that no matter who you are, you can significantly change your level of talent.
ASE_9	I can change my basic level of ability considerably.
